# Laboratory-based evaluation of the 4^th^-generation Alere^TM^ HIV Combo rapid point-of-care test

**DOI:** 10.1371/journal.pone.0298912

**Published:** 2024-02-23

**Authors:** Alice Manjate, Charlotta Nilsson, Maria Axelsson, Sarah Lindroth, Desiree Sirbu, Jahit Sacarlal, Sören Andersson, Magnus Unemo

**Affiliations:** 1 Faculty of Medicine and Health, School of Medical Sciences, Örebro University, Örebro, Sweden; 2 Faculdade de Medicina, Departamento de Microbiologia, Universidade Eduardo Mondlane, Maputo, Mozambique; 3 Public Health Agency of Sweden, Stockholm, Sweden; 4 Department of Laboratory Medicine, Division of Clinical Microbiology, Karolinska Institutet, Stockholm, Sweden; 5 Faculty of Medicine and Health, Department of Laboratory Medicine, WHO Collaborating Centre for Gonorrhoea and Other STIs, Microbiology, Örebro University, Örebro, Sweden; 6 Institute for Global Health, University College London (UCL), London, United Kingdom; Harar Health Science College, ETHIOPIA

## Abstract

**Background:**

Mozambique is a high-prevalence country for HIV and early detection of new HIV infections is crucial for control of the epidemic. We aimed to evaluate the accuracy of the 4^th^-generation rapid diagnostic test (RDT) Alere^TM^ HIV Combo in detecting acute and seroconverted HIV-infection, among sexually-active women attending three clinical health centers in Maputo, Mozambique.

**Methods:**

Women aged 14–55 years (n = 920) seeking care at the Mavalane Health Area, Maputo (February 2018-January 2019) were included, and blood specimens sampled. Sociodemographic and sexual behavior data were collected. Point-of-care HIV testing was performed using Alere Determine^TM^ HIV-1/2 and Uni-Gold^TM^ HIV-1/2. All samples were also tested using Enzygnost® HIV Integral 4 and Innotest® HIV Antigen mAb in laboratory. The 4^th^-generation RDT Alere^TM^ HIV Combo was evaluated on serum samples in the laboratory. Finally, Innotest® HIV Antigen mAb, Enzygnost® HIV Integral 4 (Ag/Ab), and HIV RNA quantification acted as gold standard assays in the evaluation of Alere^TM^ HIV Combo test for HIV antigen detection (in clinical samples and in three HIV-1 seroconversion panels).

**Results:**

The antibody component of the 4th generation Alere^TM^ HIV Combo RDT demonstrated a sensitivity and specificity of 100% examining clinical samples. However, the test did not detect HIV p24 antigen in any clinical samples, while Innotest® HIV Antigen mAb, verified by Enzygnost® HIV Integral 4 (Ag/Ab) and/or HIV RNA quantification, detected HIV antigen in six clinical samples. Furthermore, the Alere^TM^ HIV Combo RDT had a low sensitivity in the detection of HIV p24 antigen in seroconversion panels. The HIV prevalence among the examined women was 17.8%.

**Conclusions:**

The 4^th^-generation RDT Alere^TM^ HIV Combo showed similar sensitivity to the 3^rd^-generation RDTs to detect seroconverted HIV-infections. However, the sensitivity for detection of HIV p24 antigen and diagnosing acute HIV infections, before seroconversion, was low. There is an urgent need to develop and evaluate simple and affordable POC tests with high sensitivity and specificity for diagnosing individuals with acute HIV infection in resource-limited settings with high HIV prevalence.

## Introduction

The global HIV/AIDS epidemic remains one of the most serious health and development challenges in the world [1]. In 2021, approximately 1.5 million new cases of HIV infection in the adult population were estimated and about 650,000 people died from AIDS-related illnesses worldwide [1]. In Africa with two-thirds of the global population living with HIV, HIV infection is the second leading cause of death. East and Southern Africa are the regions most affected by HIV, covering approximately 45% (670,000) of new HIV infections worldwide and 54% (20.6 million) of people living with HIV worldwide [1,2]. In sub-Saharan Africa, women and girls are most affected with 63% of all new HIV infections. Among 15–24 year olds, a woman was twice as likely to be living with HIV compared to a man in 2021 [3].

Mozambique is among the nine countries most affected by HIV in Southern Africa, with a prevalence estimated at 12.4% in the adult population, being higher among women (15.4%) than men (9.5%) [4,5]. The number of people living with HIV is estimated at about 1.5 million and about 40,000 deaths occur annually from HIV-related diseases [6]. In 2019, Maputo Province had an estimated higher prevalence of 15.4% in the population of reproductive age [7]. Mozambique is also bordering the countries with the highest prevalence rates in the region (and, thus, in the world), such as Eswatini with 27.4%, Lesotho 25.0%, Zimbabwe 11.6% and South Africa 19.0% in 2020 [4,8].

HIV testing is crucial for HIV prevention, detection and care of people living with HIV, as well as for epidemiological counter measures [9–11]. The US Centers for Disease Control and Prevention’s (CDC) recommendation for HIV testing in the US is to screen with a 4^th^-generation immunoassay that detects both HIV-1/2 antigens and antibodies and, for reactive samples, a complementary immunoassay that differentiates HIV-1 antibodies from HIV-2 antibodies should be used [12]. However, this testing algorithm is not affordable for many countries with limited resources, such as Mozambique, since these laboratory-based tests are expensive and require laboratories as well as appropriate equipment and well-trained laboratory staff. Instead, in resource-poor countries the World Health Organization (WHO) recommends the use of HIV rapid diagnostic tests (RDTs), which can be used at the point of care (POC), for HIV screening and diagnosis, because of the simplicity, low cost and rapidity in providing results of these RDTs [13]. In 2015, the WHO published HIV-stratified testing guidelines for countries with HIV prevalence below and above 5%, respectively, recommending the sequential use of up to three different RDTs for the final diagnosis of HIV. These guidelines stipulated that each of the three RDTs must have a sensitivity of ≥99% and specificity of ≥98% [13]. Mozambique has, in accordance with the WHO HIV testing guidelines [13,14], implemented a national strategy for HIV screening based on 3^rd^-generation RDTs that rely on antibody detection only.

For diagnosis of acute HIV infection, the relatively expensive laboratory-based tests for HIV RNA and the anti-HIV/p24 antigen combination assays are the most sensitive [11,15]. Unfortunately, RDTs that detect HIV antigen (or a combination of HIV antigen and antibodies), i.e. 4^th^-generation HIV RDTs, have been less common, especially in many low-income settings [16] The earlier introduced Determine HIV-1/2 Ag/Ab Combo (Orgenics, Ltdof Yavne, Israel) had very low sensitivity in the detection of HIV (p24) antigen in clinical settings [17–21]. This assay has been replaced by the 4^th^-generation CE-marked Alere^TM^ HIV Combo test (Alere Medical Co. Ltd, Matsudo-shi, Japan), which has demonstrated widely variable sensitivity (0.93–95%) but high specificity (98.73–99%) in diagnosing acute infection in different study populations [22–24]. The genetic variability of HIV and differences in prevalence may influence the sensitivity of 4^th^-generation HIV RDTs, posing a challenge for the detection of acute HIV infection in different settings [25–27]. An HIV RDT allowing accurate identification of recently infected individuals at the POC would be very valuable, especially in the context of Mozambique where women of reproductive age have been the most affected [7]. This could facilitate access to immediate antiretroviral treatment (ART) reducing the HIV transmission in general [28], including reducing the vertical transmission from mother to child.

The aim of the present study was to evaluate the performance of the 4^th^-generation RDT Alere^TM^ HIV Combo for detection of acute and seroconverted HIV infection among sexually-active women attending three clinical health care centers in Maputo, Mozambique.

## Material and methods

### Study settings and population

This prospective cross-sectional study was carried out at three basic health centers (1° de Junho, 1° de Maio, and Polana Caniço) in the Mavalane Health area in Maputo Province from February 2018 to January 2019. The three health centers are located in the most populated areas of Maputo Province, with an estimated total population of 3.4 million in 2021. These health centers provide primary health care to inhabitants of different neighbourhoods of Maputo Province, especially those living in the surrounding areas [29]. A previous study conducted in these communities showed a high HIV prevalence rate (unpublished data), which motivated the selection of these sites for the present study. After obtaining written informed consent, 920 non-pregnant sexually-active women aged 14–55 years were enrolled, independent on their HIV status (if this was known for them).

### Recruitment and sampling of study participants

Women who consecutively attended their first clinical appointment during the study period at the cervical cancer screening, family planning, and sexual health clinics were recruited from these three health centers located in urban (1° de Maio), suburban (Polana Caniço), and rural (1° de Junho) areas in Maputo Province. Exclusion criteria were pregnancy, current antibiotic use, menstruation at the time of the visit and use of vaginal douches in the last 7 days. The women were invited to participate in the study by a member of the research team after explaining the objectives of the study and all participants signed informed consent prior to their inclusion in the study, in accordance with international ethical principles [30]. Subsequently, blood samples and sociodemographic information were collected. A structured questionnaire was used by a community health worker to collect variables such as age, schooling, marital status, place of residence, sexual behavior and age of onset of sexual activity.

### HIV diagnostic testing using 3^rd^-generation HIV RDTs at POC

HIV testing at the POC was performed according to the national standard sequential testing algorithm [31–33], i.e., with the minor modification that venous whole blood samples were used instead of finger prick samples. Accordingly, whole blood samples (10 ml) were collected by Vacutainer, by trained maternal health nurses. In the national algorithm, Alere Determine^TM^ HIV-1/2 (Alere Connected Health Ltd, Israel) is used as a screening assay of approximately 50 μl whole blood and Uni-Gold^TM^ HIV-1/2 (Trinity Biotech, Ireland) is used for confirmation. If the Alere Determine^TM^ HIV-1/2 test is negative, the patient is considered HIV-negative. Patients are considered HIV-infected if both assays are reactive. In indeterminate cases, the algorithm recommends collecting a new sample and repeating the algorithm. If it remains undetermined, a new test is scheduled for 4 weeks later. In the present study, this national standard sequential testing was performed, however, samples with indeterminate results, i.e., reactive using Alere Determine^TM^ HIV-1/2 followed by a non-reactive Uni-Gold HIV, were tested using the Enzygnost HIV Integral 4 assay (Siemens Healthcare Diagnostics Products GmbH, Germany), which was used to test all samples at the laboratory (see below), or tested again after three months, at which time the patient was requested to return for repeated sampling and testing. HIV-positive cases were referred to the clinic for follow up and treatment.

### Specimens for HIV testing at the laboratory

The remainants of the whole blood samples were transported daily to the Microbiology Laboratory of the Faculty of Medicine of the Eduardo Mondlane University in Maputo, in a thermal box with ice packs. Whole blood samples were centrifuged at 8000 rpm for 10–15 min and sera samples aliquoted in duplicate. One aliquot was stored at -20°C and the other at -70°C, until thawed once prior to analysis. In addition to the clinical samples, three HIV-1 seroconversion panels (75018, 73698 and 65389 from ZeptoMetrix™ LLC, Franklin, United States) were used to further evaluate the Alere^TM^ HIV Combo RDT, especially for the detection of acute HIV infection. Performance data for six HIV antibody and/or HIV antigen tests were provided for each seroconversion panel by the supplier (S1–S3 Tables).

### Evaluation of the Alere^TM^ HIV Combo rapid diagnostic test and HIV reference testing at laboratory

At the laboratory, all sera samples were tested in parallel using the 4^th^-generation RDT Alere^TM^ HIV Combo (Alere Medical Co. Ltd) and two HIV reference ELISAs. The reference ELISAs, Innotest HIV Antigen mAb (Fujirebio Europe, Belgium) and Enzygnost HIV Integral 4 (Siemens Healthcare Diagnostics Products GmbH, Germany), were considered the gold standard serological assays for the detection of HIV p24 antigen and HIV antibodies, respectively.

All assays were performed and interpreted according to the manufacturers’ instructions and all discrepant results were repeated in duplicate. The main characteristics of all the HIV serological assays used in the present study are summarized in S4 Table.

### HIV RNA quantification

Samples reactive by the HIV antigen assay (Innotest HIV Antigen mAb) were further confirmed by quantification of HIV-1 RNA (COBAS Ampliprep/COBAS TaqMan HIV-1 test, v2.0; Roche Diagnostics GmbH, Germany) at the Molecular Biology Laboratory of the National Institute of Health, Maputo. Testing was performed using 1 mL of undiluted serum and, in some cases, diluted according to the manufacturer’s instructions. The minimum and maximum limits of detection were 20 copies/ml and 10,000,000 copies/mL, respectively.

### Acute HIV infection definition

Acute HIV infection is the earliest phase of HIV infection, immediately after viral acquisition and before seroconversion, when usually both HIV RNA and p24 antigen are present in the body fluids of the infected individual [34,35]. In the present study, we defined acute HIV infection as HIV antigen positive, HIV RNA positive, and HIV antibody negative.

### Statistical analysis

All statistical analyses were performed using SPSS v.20 (SPSS Inc. IBM Corp., Armonk, New York, USA). Cross tabulation was used to calculate sensitivity, specificity, positive predictive value (PPV) and negative predictive value (NPV) for the 4^th^-generation RDT Alere^TM^ HIV Combo (Alere Medical Co. Ltd). Descriptive statistics was used to show the distribution of sociodemographic characteristics and HIV status.

### Ethical approval

Ethical approval from the National Bioethics Commission for Health of Mozambique was obtained before the start of the study, with reference 405/CNBS/2014, and renewed to the current reference 111/CNBS/22. Notable, all participating women were subject to informed written consent. In addition to informed written consent of the participant, written parental consent was also collected for those under 18 years of age.

## Results

### Study population

In total, 1081 participants were recruited, however, 14.9% (161/1081) of these participants were excluded due to insufficient sample volume for testing with all assays. Accordingly, the samples of 920 participants were analysed in the present study (S5 Table). The main sociodemographic data for the study participants are summarized in Table 1.

**Table 1:** Sociodemographic characteristics of study participants.

Characteristics	Total (n = 920)	Percentage (%)	HIV positive (%)
**Age—Years, Mean (SD)**	29.7 (9.7)		
**Age–Years** 14 15–19 20–24 25–29 30–34 35–39 40–44 45–49 50–55	1 113 256 140 131 123 67 55 34	0.1 12.3 27.8 15.2 14.2 13.4 7.3 6.0 3.7	0 2.7 9.0 17.1 21.4 38.2 23.9 27.3 23.5
**Place of current residence** URBAN AREA RURAL AREA SUBURBAN AREA OTHERS	61 516 341 2	6.6 56.1 37.1 0.2	4.9 20.7 15.5 50.0
**Marital status** SINGLE MARRIED DIVORCED COHABITING WIDOW	454 124 23 298 21	49.3 13.5 2.5 32.4 2.3	13.0 14.5 30.4 22.1 66.7
**Age of first intercourse—year** <12 12–14 15–17 18–20 21–23 24–26 >26 DO NOT REMEMBER	1 55 481 312 37 8 1 25	0.1 6.0 52.3 33.9 4.0 0.9 0.1 2.7	0 34.5 17.7 14.7 8.1 0 0 44.0
**Level of education** PRIMARY SCHOOL SECONDARY SCHOOL HIGHER EDUCATION NONE	119 483 300 18	12.9 52.5 32.6 2	37.0 16.1 10.3 61.1

N: Number; SD: Standard deviation.

Briefly, the median (mean) age of the included participants was 27 (29.7) years. The majority (56.1%, 516/920) of participants lived in a rural area and 49.3% (454/920) were single. Two percent (18/920) had no school education, while 32.6% (300/920) had attended higher education (Table 1). Mean age of sexual debut was 17.2 years old (SD±2.1).

### HIV prevalence

The overall HIV prevalence was 17.8% (164/920), i.e., based on testing of venous whole blood with the 3^rd^-generation RDTs Determine HIV-1/2 and Uni-Gold HIV. In addition, all samples were confirmed with the reference test Enzygnost HIV Integral 4. The highest HIV prevalence was observed in the age group of 35–39 years (38.2%) and in those without academic education. The HIV prevalence differed substantially between places of residence, with women living in rural areas being the most affected with a prevalence of 20.7% and women living in urban areas with the lowest HIV prevalence (4.9%) (Table 1).

### Performance of the 4^th^-generation rapid diagnostic test Alere^TM^ HIV Combo

#### Clinical specimens

The antibody-component of the Alere^TM^ HIV Combo RDT was reactive for all (100%, 164/164) seroconverted HIV infections detected in venous whole blood samples by the 3^rd^-generation RDTs Determine HIV-1/2 and Uni-Gold HIV plus the 4^th^-generation Enzygnost HIV Integral 4 test. Furthermore, no false-reactive samples, indeterminate samples or invalid results were observed (Table 2). Accordingly, the sensitivity, specificity, NPV and PPV of the antibody-component of the Alere^TM^ HIV Combo were all 100%.

**Table 2:** 4^th^-generation rapid diagnostic test (RDT) Alere^TM^ HIV Combo compared to the 3^rd^-generation RDTs Determine HIV-1/2 and Uni-Gold HIV plus the reference 4^th^-generation test Enzygnost HIV Integral 4.

**Alere^TM^ HIV Combo**	**National HIV testing + Enzygnost HIV Integral 4**			
		Positive	Negative	Total
	Positive	164	0	164
	Negative	0	756	756
	Total	164	756	920

However, no samples were reactive for HIV p24 antigen using the 4^th^-generation RDT Alere^TM^ HIV Combo. This was in contrast to the Innotest HIV Antigen mAb test that identified 1.6% (15/920) of samples as HIV p24 antigen reactive, of which six samples were confirmed by the reference test Enzygnost HIV Integral 4 and/or HIV-1 RNA PCR, with viral loads ranging from 79 to 37,586 copies/ml. Accordingly, nine samples (0.98%, 9/920) were considered to be false positive using the Innotest HIV Antigen mAb ELISA, *i*.*e*. the samples were negative with both Enzygnost HIV Integral 4 and/or HIV-1 RNA PCRs. Only one of the six confirmed samples (86 copies/ml) was defined as a possible acute HIV infection (0.11%, 1/920) because the other five samples showed also HIV antibody reactivity using the RDTs Alere^TM^ HIV Combo, Determine HIV-1/2 and Uni-Gold HIV, as well as they were reactive using the reference Enzygnost HIV Integral 4 test. Accordingly, these individuals had seroconverted. Furthermore, we defined this single case as a ‘possible’ acute HIV infection because the HIV viral load was very low and Enzygnost HIV Integral 4 was negative, i.e. did not detect any HIV antigen or antibodies (Table 3).

**Table 3:** Samples non-reactive for HIV antigen using Alere^TM^ HIV Combo test but reactive for HIV antigen using Innotest HIV Antigen mAb and HIV-1 RNA PCR.

Samples ID	Alere^TM^ HIV Combo	Innotest HIV Antigen mAb	HIV RNA PCR (Copies/ml)	Enzygnost (Ag/Ab)	Determine RDT	Uni-Gold RDT
479	Ag-	Ag+	86	Nonreactive	Ab-	Ab-
110	Ag-	Ag+	36925	Reactive	Ab+	Ab+
164	Ag-	Ag+	373515	Reactive	Ab+	Ab+
290	Ag-	Ag+	37586	Reactive	Ab+	Ab+
345	Ag-	Ag+	1664	Reactive	Ab+	Ab+
1043	Ag-	Ag+	79	Reactive	Ab+	Ab+

Ag-: Non-reactive for HIV antigen; Ab-: Non-reactive for HIV antibodies; RDT: Rapid diagnostic test.

#### HIV-1 seroconversion panels

The performance of the Alere^TM^ HIV Combo RDT was also analysed using three commercial acute HIV-1 infection seroconversion panels.

In panel number 75018, the Alere^TM^ HIV Combo RDT was HIV p24 Ag-positive at the same time point as the 3rd generation Oraquick Advance Rapid HIV-1/2 Ab test (detecting only HIV antibodies), and seven days after the Abbott Architect HIV Ag/Ab Combo, the Zeptometrix HIV P24 Ag and the Bio-Rad BioPlex 2200 HIV-1 Ag-Ab 5th generation and 11 days after the most sensitive reference test detected HIV RNA (Gen-probe Procleix HIV-1/HCV assay). However, five days later, the Alere^TM^ HIV Combo RDT became reactive for HIV antibodies. The seroconversion interval (from testing initially positive for Ag until testing positive for Ab) using the Alere^TM^ HIV Combo RDT was ≤5 days (S2 Table).

In panel 73698, the Alere^TM^ HIV Combo RDT detected HIV p24 antigen almost two weeks before the Oraquick Advance Rapid HIV-1/2 Ab test detected any HIV antibodies (which were detected nearly one week earlier with the Alere^TM^ HIV Combo RDT), and at the same time point as the Abbott Architect HIV Ag/Ab Combo and Abbott™ HIV-1 p24 Ag tests. The Alere^TM^ HIV Combo RDT was also positive one week earlier than the Bio-Rad HIV-1 Western blot, but seven days later than the most sensitive reference test detected HIV RNA (COBAS® AmpliPrep/COBAS®/TaqMan® HIV-1). The seroconversion interval using the Alere^TM^ HIV Combo RDT was ≤7 days (S3 Table).

In the third panel, 65389, the Alere^TM^ HIV Combo RDT did not detect any samples that were only positive for HIV antigen. From negative samples it immediately moved to dual positivity reactivity (reactive for both antigens and antibodies at the same time). However, the Oraquick Advance Rapid HIV-1/2 Ab test remained non-reactive in all samples. Alere^TM^ HIV Combo RDT dual reactivity occurred five days after Abbott Architect HIV Ag/Ab Combo and Abbott HIV-1 p24 Ag tests, seven days after Coulter HIV-1 p24 Ag test, and three weeks after reactivity of the most sensitive reference test (Chiron HIV-1 bDNA) (S4 Table).

## Discussion

The introduction of RDTs detecting HIV antibodies has substantially expanded HIV diagnosis in sub-Saharan Africa due to their simplicity of performance and rapid provision of results, allowing for large-scale testing of the populations, even at the POC beyond more equipped health care facilities, by personnel with limited training. However, the detection of acute HIV infection, i.e., prior to seroconversion, remains a challenge in this region of Africa and similar settings internationally, due to the lack of appropriate RDTs detecting also HIV antigens [12].

In this study, we evaluated the 4^th^-generation Alere^TM^ HIV Combo RDT for its potential to improve the detection of acute HIV infection in a POC setting in Mozambique. In clinical samples from women in Maputo, Mozambique, the sensitivity, specificity, and the predictive value (PPV) of the HIV antibody-detection component of the 4^th^-generation Alere^TM^ HIV Combo RDT were all 100%. However, the detection of HIV p24 antigen and acute HIV infection with this assay was suboptimal in our clinical samples. The prevalence of HIV acute infection was very low in our clinical material, but it could have been slightly underestimated because HIV RNA quantification was not performed on all samples (and only on serum and not on more ideal plasma samples) due to logistical and economical reasons. Notably, acute HIV infection is defined as the period before detectable antibodies develop and includes both the stages Fiebig I, in which only HIV RNA is detectable, and Fiebig II in which HIV p24 antigen becomes detectable [36–38]. Nevertheless, the low sensitivity of the HIV antigen-detection component of the 4^th^-generation Alere^TM^ HIV Combo RDT was further confirmed in the testing of three commercial HIV-1 seroconversion panels. Other studies evaluating the 4^th^-generation Alere^TM^ HIV Combo RDT have reported sensitivities ranging from around 0.94% to 95% in the detection of HIV acute infections [11,22,24,39–44].

In general, screening for HIV p24 protein is challenging due to the short time that this core antigen can be detected in infected individuals. It has been reported that when HIV antibodies become detectable in the blood, the detection of p24 antigen can rapidly decrease [45,46]. Some authors have also reported that the high genetic diversity of the HIV-1 Gag subtype can influence the detection limit of p24 antigen tests, especially with non-B subtypes [47]. According to Bila *et al* [48], the HIV-1-C subtype predominates in Mozambique, similar to other countries in the region, which could potentially decrease the sensitivity of HIV p24 detection assays. Furthermore, in general detection of HIV-1 p24 by 4^th^-generation ELISAs, such as Enzygnost or Innotest, is less sensitive than testing for HIV RNA (viral load), as this biomarker becomes detectable before the HIV p24 antigen [49].

In the present study, the peak prevalence of HIV (38.2%) was observed in the group of 35–39 years old, being consistent with previously published data [7,50,51], but different from findings by Dias et al. [52] that described the highest odds of being HIV-positive in the group of 25–29 years old. Worryingly, in low-income countries such as Mozambique, the majority of the population lives in rural or suburban areas (slums) and with low economic status. According to Fagbamigbe *et al*. [53], these groups of women are high risk groups for HIV, as they are easily sexually harassed in exchange for money or even food products.

The limitations of our study included that the Alere^TM^ HIV Combo RDT was evaluated using serum samples by well-trained personnel in the laboratory, which may have resulted in improved results compared to using finger prick samples at the POC, and the lack of HIV RNA quantification (or HIV qualitative nucleic acid amplification test) in all samples (and only serum samples and not the more ideal plasma samples were tested), which might have resulted in missing some acute HIV infections in the Fiebig I stage. Furthermore, the women examined in the present study were not representative for the overall population of women and, accordingly, cannot be generalized to other populations, i.e., due to our study design, including non-probability sampling, and the inclusion of mainly women with urogenital complaints. The strengths included that the present study is one of very few studies carried out in Mozambique that both evaluated a 4^th^-generation RDT and determined the prevalence of HIV based on the two 3^rd^-generation RDTs Determine HIV-1/2 and Uni-Gold HIV used in the national HIV testing algorithm (which was also further evaluated by using two laboratory-based 4^th^-generation references ELISAs) among sexually-active women attending several clinical health centers in Maputo, Mozambique.

## Conclusions

The 4^th^-generation Alere^TM^ HIV Combo RDT showed high sensitivity and specificity in detecting HIV antibodies with performance similar to 3^rd^-generation RDTs but p24 antigen component did not work well. Accordingly, the sensitivity in the detection of HIV p24 antigen in clinical samples and acute HIV infection, as well as in three HIV-1 seroconversion panels was low. This indicates that the HIV p24 antigen detection using the evaluated test is not likely to add substantially to early HIV diagnosis in the current format. There is still a need for the development and evaluation of simple and affordable POC tests with high sensitivity and specificity for diagnosing individuals with acute HIV infection in resource-limited settings with high HIV prevalence. Nevertheless, acute HIV infection is rare. The HIV prevalence among sexually-active women who visited health care centers in Maputo, Mozambique, remained very high. Accordingly, improved HIV education (safe sexual behavior and reducing stigma), prevention (e.g., counselling and promoting safe sex and condom use), diagnostic testing (including tests accurately detecting both acute HIV infections and seroconverted infections), and treatment (including monitoring of treatment effects) focused on especially, but not exclusively, populations at higher risk are imperative to combat the HIV epidemic in Mozambique.

## Supporting information

S1 Fig Flowchart describing collected samples and HIV testing performed. (DOCX)

S1 Table HIV-1 seroconversion panel No. 75018. Only the Alere^TM^ HIV Combo rapid diagnostic test was performed in the present study.
(DOCX)

S2 Table HIV-1 seroconversion panel No. 73698. Only the Alere^TM^ HIV Combo rapid diagnostic test was performed in the present study.
(DOCX)

S3 Table HIV-1 seroconversion panel No. 65389. Only the Alere^TM^ HIV Combo rapid diagnostic test was performed in the present study.
(DOCX)

S4 Table List of HIV tests used in the study and their main characteristics. (DOCX)

S5 Table De-identified metadata for all participants in the present study. (XLSX)
