# Supplementary material for: Laboratory-based evaluation of the 4th-generation AlereTM HIV Combo rapid point-of-care test
Source: PLoS One. 2024 Feb 23;19(2):e0298912. doi: 10.1371/journal.pone.0298912 (PMC10889622; doi:10.1371/journal.pone.0298912)
Supplement: S1 Fig — (DOCX) [file pone.0298912.s001.docx]

**1081 venous whole blood samples collected**

**Point of care, HIV testing:**

All samples tested with:
3^rd^ generation RDTs (Alere Determine HIV-1/2 and Uni-Gold HIV-1/2)

**Sample type tested: Whole blood**

**161 samples excluded** (insufficient volume to perform all additional tests after the RDTs)

**920 samples further tested**

**Laboratory, HIV testing:**

All samples tested with:

- 4^th^ generation Alere HIV Combo RDT
- 4^th^ generation Enzygnost HIV Integral 4
- Innotest HIV Antigen mAb

**Sample type tested: Serum**

**HIV Ag positive (n=15)**

(positive test: Innotest HIV Antigen mAb)

tested with Alere HIV combo

**HIV Ab positive (n=164)**

(positive tests: 3^rd^ generation RDTs, Alere HIV Combo RDT, Enzygnost HIV Integral 4)

tested with Alere HIV combo

10 Ag Positive only

05 Ag+ Ab Positive

05 A14)

tested with Alere HIV combo

COBAS Ampliprep/COBAS TaqMan HIV-1 test, v2.0 (HIV-1 RNA quantification)

**Sample type tested: Serum**

**HIV-1 RNA detected (n=6)**

tested with Alere HIV combo

**HIV Ab positive (n=5): Seroconverted HIV infection**

**tested with Alere HIV combo**

**HIV Ab negative (n=1):**

**Acute HIV infection**

**tested with Alere HIV combo**

**S1 Figure. Flowchart describing collected samples and HIV testing performed.**RDT, rapid diagnostic test; Ab, antibody; Ag, antigen
