# Supplementary material for: Laboratory-based evaluation of the 4th-generation AlereTM HIV Combo rapid point-of-care test
Source: PLoS One. 2024 Feb 23;19(2):e0298912. doi: 10.1371/journal.pone.0298912 (PMC10889622; doi:10.1371/journal.pone.0298912)
Supplement: S1 Table — Only the AlereTM HIV Combo rapid diagnostic test was performed in the present study. (DOCX) [file pone.0298912.s002.docx]

| **S1 Table. HIV-1 seroconversion panel No. 75018. Only the Alere^TM^ HIV Combo rapid diagnostic** **test was performed in the present study.** | | | | | | | | |
| --- | --- | --- | --- | --- | --- | --- | --- | --- |
| **Date of draw** | **Gen-Probe Procleix® HIV-1/HCV assay** | **Abbott Architect HIV Ag/Ab Combo** | | **Bio-Rad HIV-1 Western blot** | **OraQuick ADVANCERapid HIV-1/2 Ab** | **Zepto-metrix HIV-1 p24 Ag** | **Bio-Rad BioPlex® 2200 Ag-Ab (5th generation)** | **Alere™ HIV Combo**  **reactivity** |
| 20-Oct-99 | Negative | 0.09 | Not tested | | Negative | <3.0 | 0.1 | Negative |
| 25-Oct-99 | Negative | 0.28 | Not tested | | Negative | <3.0 | 0.07 | Negative |
| 27-Oct-99 | Negative | 0.09 | Not tested | | Negative | <3.0 | 0.03 | Negative |
| 02-Nov-99 | Negative | 0.19 | Not tested | | Negative | <3.0 | 0.13 | Negative |
| 04-Nov-99 | Negative | 0.13 | Not tested | | Negative | <3.0 | 0.04 | Negative |
| 09-Nov-99 | Negative | 0.15 | Not tested | | Negative | <3.0 | 0.08 | Negative |
| 11-Nov-99 | Negative | 0.1 | Not tested | | Negative | <3.0 | 0.04 | Negative |
| 16-Nov-99 | Negative | 0.22 | Not tested | | Negative | <3.0 | 0.07 | Negative |
| 18-Nov-99 | Negative | 0.12 | Not tested | | Negative | <3.0 | 0.11 | Negative |
| 23-Nov-99 | Negative | 0.11 | Not tested | | Negative | <3.0 | 0.04 | Negative |
| 30-Nov-99 | Positive | 0.15 | Not tested | | Negative | <3.0 | 0.17 | Negative |
| 04-Dec-99 | Positive | 2.83 | Negative | | Negative | 6.3 | 10.00 | Negative |
| 11-Dec-99 | Positive | 42.80 | Negative | | Positive | >125 | 166.91 | Ag+ |
| 16-Dec-99 | Positive | 20.42 | GP160, P55, P40, P24 | | Positive | 29.3 | 24.49 | Ab+ |
| 18-Dec-99 | Positive | 17.99 | GP160, P55, P40, P24 | | Positive | 4.8 | 4.02 | Ab+ |
| 23-Dec-99 | Positive | 11.14 | GP160, P55, P40, P24 | | Positive | <3.0 | 0.62 | Ab+ |
| 26-Dec-99 | Positive | 8.45 | GP160, P55, P40, P24 | | Positive | <3.0 | 0.49 | Ab+ |
| 30-Dec-99 | Positive | 5.99 | GP160, P65+/-, P55,  P40, P24, P18+/- | | Positive | <3.0 | 0.29 | Ab+ |
| 02-Jan-00 | Positive | 5.38 | GP160, P65, P55, P40, P31+/-, P24, P18+/- | | Positive | <3.0 | 0.19 | Ab+ |
| 06-Jan-00 | Positive | 5.07 | GP160, P65, P55,  P40, P31, P24, P18+/- | | Positive | <3.0 | 0.12 | Ab+ |
| 09-Jan-00 | Positive | 5.64 | GP160, P65, P55,  P40, P31, P24, P18 | | Positive | <3.0 | 0.07 | Ab+ |
| 16-Jan-00 | Positive | 9.54 | GP160, P65, P55, GP41+/-, P40, P31, P24, P18 | | Positive | <3.0 | 0.12 | Ab+ |
| 20-Jan-00 | Positive | 15.37 | GP160, P65, P55, GP41+/-, P40, P31, P24, P18 | | Positive | <3.0 | 0.09 | Ab+ |
