# Supplementary material for: Laboratory-based evaluation of the 4th-generation AlereTM HIV Combo rapid point-of-care test
Source: PLoS One. 2024 Feb 23;19(2):e0298912. doi: 10.1371/journal.pone.0298912 (PMC10889622; doi:10.1371/journal.pone.0298912)
Supplement: S2 Table — Only the AlereTM HIV Combo rapid diagnostic test was performed in the present study. (DOCX) [file pone.0298912.s003.docx]

| S2 Table. HIV-1 seroconversion panel No. 73698. Only the Alere^TM^ HIV Combo rapid diagnostic test was performed in the present study. | | | | | | | |
| --- | --- | --- | --- | --- | --- | --- | --- |
| Date of draw | **Roche Cobas Amplicor HIV-1 monitor** | **Abbott Architect  HIV Ag/Ab Combo** | **Bio-Rad HIV-1  Western blot** | **OraQuick  ADVANCERapidHIV-1/2 Ab** | **Coulter HIV-1  p24 Ag** | **Abbott HIV-1 p24 Ag** | **Alere™ HIV Combo Rapid Test** |
| 25-Sep-99 | Negative | 0.08 | Not tested | Negative | 0.213 | 0.276 | Negative |
| 27-Sep-99 | Negative | 0.14 | Not tested | Negative | 0.160 | 0.328 | Negative |
| 02-Oct-99 | Negative | 0.09 | Not tested | Negative | 0.191 | 0.241 | Negative |
| 04-Oct-99 | Negative | 0.11 | Not tested | Negative | 0.181 | 0.293 | Negative |
| 09-Oct-99 | Negative | 0.10 | Not tested | Negative | 0.191 | 0.259 | Negative |
| 11-Oct-99 | Negative | 0.10 | Not tested | Negative | 0.181 | 0.293 | Negative |
| 16-Oct-99 | 2000 | 0.13 | Not tested | Negative | 0.245 | 0.259 | Negative |
| 18-Oct-99 | 44700 | 0.53 | Negative | Negative | 0.511 | 0.517 | Negative |
| 23-Oct-99 | >750000 | 50.26 | Negative | Negative | 28.723 | 14.293 | Ag+ |
| 28-Oct-99 | >750000 | 167.70 | Negative | Negative | 34.043 | 34.483 | Ag+ |
| 30-Oct-99 | >750000 | 53.66 | GP160, P55+/-, P24 | Negative | 34.043 | 9.655 | Ag+/Ab+ |
| 04-Nov-99 | 143000 | 28.35 | Indeterminate | Positive | 2.053 | 0.810 | Ab+ |
| 06-Nov-99 | Not tested | 30.20 | Indeterminate | Positive | 0.638 | 0.483 | Ab+ |
