# Supplementary material for: Laboratory-based evaluation of the 4th-generation AlereTM HIV Combo rapid point-of-care test
Source: PLoS One. 2024 Feb 23;19(2):e0298912. doi: 10.1371/journal.pone.0298912 (PMC10889622; doi:10.1371/journal.pone.0298912)
Supplement: S3 Table — Only the AlereTM HIV Combo rapid diagnostic test was performed in the present study. (DOCX) [file pone.0298912.s004.docx]

| S3 Table. HIV-1 seroconversion panel No. 65389. Only the Alere^TM^ HIV Combo rapid diagnostic test was performed in the present study. | | | | | | | |
| --- | --- | --- | --- | --- | --- | --- | --- |
| Date of draw | **Chiron HIV-1 bDNA** | **Abbott Architect HIV Ag/Ab Combo** | **Cambridge Biotech HIV-1 Western blot** | **OraQuick  ADVANCERapid HIV-1/2 Ab** | **Coulter HIV-1 p24 Ag** | **Abbott HIV-1 p24 Ag** | **Alere™ HIV Combo** |
| 12-nov-97 | 157 | 0.10 | Negative | Negative | 0.08 | 0.51 | Negative |
| 14-nov-97 | <50 | 0.07 | Negative | Negative | 0.08 | 0.38 | Negative |
| 19-nov-97 | 69 | 0.08 | Negative | Negative | 0.08 | 0.51 | Negative |
| 21-nov-97 | 221 | 0.11 | Negative | Negative | 0.08 | 0.38 | Negative |
| 26-nov-97 | 33 740 | 0.37 | Negative | Negative | 1.05 | 0.63 | Negative |
| 28-nov-97 | 101 800 | 1.02 | Negative | Negative | 4.02 | 1.39 | Negative |
| 03-dec-97 | >500 000 | 15.17 | Negative | Negative | 50.47 | 14.81 | Ag+/Ab+ |
| 05-dec-97 | >500 000 | 49.99 | P24, P160 (VF) | Negative | 59.73 | >25.32 | Ag+/Ab+ |
|  | | | | | | | |
|  | | | | | | | |
