# Supplementary material for: Laboratory-based evaluation of the 4th-generation AlereTM HIV Combo rapid point-of-care test
Source: PLoS One. 2024 Feb 23;19(2):e0298912. doi: 10.1371/journal.pone.0298912 (PMC10889622; doi:10.1371/journal.pone.0298912)
Supplement: S4 Table — (DOCX) [file pone.0298912.s005.docx]

| **p, protein; gp, glycoprotein** | | | | | |
| --- | --- | --- | --- | --- | --- |

| **Rapid diagnostic tests** | | **Detection** | **Assay type** | | **Clinical sample (volume needed)** |  |  |
| --- | --- | --- | --- | --- | --- | --- | --- |
| Alere^TM^ HIV Combo | | HIV-1 p24 and gp41 and HIV-2 gp36 antibodies; and p24 antigen | Immunochromatographic lateral flow | | Plasma, serum, whole blood (50 μl) |  |  |
| Alere Determine^TM^ HIV-1/2 | | HIV-1 gp41, HIV-2 gp36, recombinant protein and synthetic peptide antibodies | Immunochromatographic lateral flow | | Plasma, serum, whole blood (50 μl) |  |  |
| Uni-Gold^TM^ HIV-1/2 | | HIV-1 gp41, gp120, HIV-2 gp36 and recombinant protein antibodies | Immunochromatographic lateral flow | | Plasma, serum, whole blood (50 μl) |  |  |
| **Laboratory-based enzyme immunoassays** | **Detection** | | | **Assay type (clinical sample/volume)** | | | |
| Innotest HIV Antigen mAb | HIV p24 antigen using human polyclonal antibodies | | | Sandwich test that captures HIV p24 antigen present in sample (serum or plasma/100 μl) | | | |
| Enzygnost HIV Integral 4 | Antibodies against a mixture of recombinant proteins including HIV-1/0 gp41 and HIV-2 gp36; and p24 antigen using two monoclonal antibodies (mouse). | | | Combined sandwich test that captures HIV antibodies and p24 antigens in sample (serum or plasma/100 μl) | | | |

| **S4 Table. List of HIV tests used in the study and their main characteristics.** |
| --- |
